# Using virtual reality as a replacement for hospital tours during residency interviews

**DOI:** 10.1080/10872981.2020.1777066

**Published:** 2020-06-04

**Authors:** Juan-Pablo Zertuche, Jeremy Connors, Aaron Scheinman, Neil Kothari, Kristin Wong

**Affiliations:** a Departments of Medicine and Pediatrics, Rutgers New Jersey Medical School, Newark, NJ, USA; b Designated Institutional Official, and Associate Dean of Graduate Medical Education, Department of Medicine, Rutgers New Jersey Medical School, Newark, NJ, USA

**Keywords:** Hospital tours, virtual reality, residency training, medical facility tours, virtual interviews, applicant interview day

## Abstract

**Background:**

Residency programs invest a significant amount of time and resources on the recruitment process, and maintaining efficiency and cost-effectiveness are very important. Virtual Reality (VR) has become an adaptive substitute for ‘real life’ experiences and its use during the interview season could help save time and resources.

**Objective:**

With the intention to maximize the interview day and provide a cost-effective alternative to facility tours, a Med-Peds residency training program introduced a VR tour of their children’s hospital during recruitment.

**Design:**

The Med-Peds program replaced an in-person facility tour of the children’s hospital with a VR tour. Applicants were asked to complete an anonymous, voluntary survey on their VR experience at the end of the interview season, and rank features of the interview day in order of importance.

**Results:**

There were 33 respondents out of 54 interviewees. Approximately two thirds (63–66%) agreed that VR was non-inferior and superior to in-person facility tours, and that the use of VR had a favorable impact on their perception of the program. However, almost 50% of the applicants had some difficulty using VR technology.

**Conclusion:**

Use of VR facility tours as an alternative to in-person tours of affiliate training facilities during a residency interview day is a viable and innovative option that can save time and money and favorably impact the applicant’s impression of the program. More research is necessary to assess whether VR tours can replace in-person tours at the main teaching site, however, while social distancing measures are in place, VR tours may become necessary for programs moving forward.

**Abbreviations:**

Med-Peds: Internal Medicine-Pediatrics; VR: Virtual Reality; AAMC: Association of American Medical Colleges; IRB: Institutional Review Board

## Introduction

Residency programs invest a significant amount of time and resources on the recruitment process, with a recent surgery publication estimating approximately 100,000 USD ± 87,000 per interview season for university-based programs. [1] In an era of increasing focus on cost containment, such lavish spending has generated calls for making the process more cost efficient. Applicants are also financially challenged during the recruitment season. Given the time and money required for travel, lodging, interviewing, and the application itself, there is further impetus to explore avenues to limit costs on both sides. With the onset of the COVID-19 pandemic, residency programs are also now forced to consider how to provide traditional, in-person interviews to applicants in a socially distanced or virtual method. With the interview day playing such a significant role for residents and programs, it is imperative to focus on providing applicants with information they find most useful, but in a safe and cost-effective manner.

Many university-based residency training programs are faced with providing applicants an accurate portrayal of what their residency experience will be like, but are challenged with the fact that their residents participate in clinical rotations at multiple teaching hospitals. These hospitals include a combination of private, public and military affiliated sites, and often result in residents being required to travel several miles between sites. During the interview season, programs choose to either provide tours of all hospitals or to only provide tours of the primary teaching site and describe the alternate teaching sites. COVID-19 is dramatically changing these traditional strategies as national organizations like the Association of American Medical Colleges (AAMC) are strongly encouraging that interviews and tours be held ‘in a virtual setting – either by phone or video conferencing.’ [2]

Virtual Reality (VR) is a technology that has emerged as a cost-effective and impactful tool for use in medical education. By immersing the user in a computer generated real or artificial three-dimensional environment, it has become an adaptive substitute for ‘real life’ experiences. The primary component of a VR experience typically includes a motion sensitive screen built into a goggle or headset. The user then wears the headset and immerses themselves in a 360-degree visual environment. By steering the motion sensitive device with head movements, they are able to navigate the virtual environment as if they were actually there. The 3D-environment can range from static images to full-motion video and can have audio associated with it. The technology has become less expensive since its introduction and is now more widely used in different industries ranging from medicine, gaming and education, to tourism and sales. [3]

Its use and acceptance in the medical field has been rapidly growing, and it has become an emerging educational strategy with the potential of transforming health professional education. Some studies show that VR improves post-intervention knowledge scores and health professionals’ cognitive skills compared to traditional learning. [4] During the 2015–2016 interview season, Crawford et al used VR as an insightful interview method through interactive gaming, in which they were able to observe the applicants’ communication abilities, subtle personality traits and teamwork skills. [5]

With the intention to maximize the interview day and provide a cost-effective alternative to off-site hospital tours, the Internal Medicine-Pediatrics (Med-Peds) Residency Program at Rutgers New Jersey Medical School introduced a VR tour of its off-site children’s hospital during recruitment and reviewed feedback from the applicants. While this study was performed prior to COVID-19 pandemic and the new AAMC recommendations, we take the opportunity to discuss the implications of VR technology on the future of the applicant interview process.

## Methods

The Med-Peds residency training program interviewed 54 applicants during the 2018–2019 interview season. In previous years, applicants would receive an in-person hospital tour of the primary teaching site and then be shuttled to the alternate teaching site where an in-person tour of the children’s hospital would follow. Applicants would then be shuttled back to the primary teaching hospital before finishing the interview day. Transportation costs between hospitals totaled 2,520 USD over 7 interview days, and total travel time between both hospitals accounted for 1 hour per interview day, not including the tours themselves. To minimize travel time and costs, the program introduced VR as a substitute for the alternate site, in-person, hospital tour as part of the 2018–2019 interview, but maintained the in-person hospital tour of the primary hospital. Both tours included several points of interest, ranging from the intensive care units to the cafeteria, but removed the travel required to go from site to site.

To create the VR experience of the alternate teaching site, a VR headset utilizing Google Cardboard and the Google Cardboard application (app) for VR were used. Photos of the alternate teaching site were taken with a 360-degree camera prior to the start of the recruitment season. Applicants were asked to download the free app as well as thirteen 360-degree images of several points of interest to their smart phones about 1–2 weeks prior to their interview day. On the interview day, applicants were then given a cardboard-constructed goggle that can house the user’s smart phone and be worn over the eyes with an elastic headband. If the applicant did not have a smart phone, they were able to borrow one from a current resident or faculty member. The app pulled the 360-degree images from the user’s smart phone which appeared three-dimensional when viewed inside of the headset. As the user turns the head left, right, up, and down, the viewer’s perspective moves accordingly, allowing for 360-degree visualization of the virtual environment. A faculty member guided the applicants on a VR tour of the children’s hospital, providing an explanation of the different points of interest, while the applicants remained seated at the primary site. The applicants were encouraged to browse freely through the images and ask questions. Afterwards, they were given the Google Cardboard set to take home and could access the images for future reference. The cardboard goggles had a purchase price of 3 USD/goggle and a total cost of 175. USD The entire VR tour experience took approximately 20–30 minutes.

After the rank list submission was due in February and before Match Day 2019, applicants were invited to complete an anonymous, voluntary survey about their interview experience with the program. The survey included specific statements about the use of VR and its potential advantages, and asked applicants to indicate their level of agreement or disagreement. (Table 1) The applicants were also given the opportunity to share their thoughts on VR as a tool during the interview day and were asked to rank the Virtual Reality tour and 10 other interview features in order of importance. (Table 2)

**Table 1.** Survey statements.

Survey Statements	
I.	I believe Virtual Reality is a comparable substitute for in-person facility tours.
II.	I prefer Virtual Reality tours over in-person facility tours.
III.	The use of Virtual Reality had a favorable impact on my perception of the program.
IV.	I had difficulty using the Virtual Reality goggles.
V.	I would like to see more programs using Virtual Reality in the interview day.
VI.	It was helpful to be able to review the facility pictures after the interview day.
VII.	I appreciate being able to use the provided Virtual Reality head set for other purposes since the interview day.

**Table 2.** Features of interview day ranked by importance to applicants (1: most important, 11: least important).

Rank Importance (by applicants)	Interview Day Features
1	Time with residents and overall resident culture
2	Time with MedPeds Program Director
3	Time with Interviewers
4	Pre-interview dinner
5	Time with core leadership (Pediatrics PD, Medicine PD, Faculty, etc.)
6	Office staff availability and friendliness
7	Academic experience (seeing Grand Rounds, morning reports, etc.)
8	Facility tour of University Hospital
9	Virtual reality tour of Newark Beth Israel
10	Quality of food on interview day
11	Other

The survey was distributed via email using Qualtrics as a platform for anonymous reply. It was completed by applicants between February and March 2019 and weekly reminders were sent to non-respondents until the survey was closed on Match Day, March 15th, 2019. The responses were analyzed using Excel. The study protocol was approved as an exempt review by the Rutgers Health Sciences IRB in Newark.

## Results

**Figure 1.**
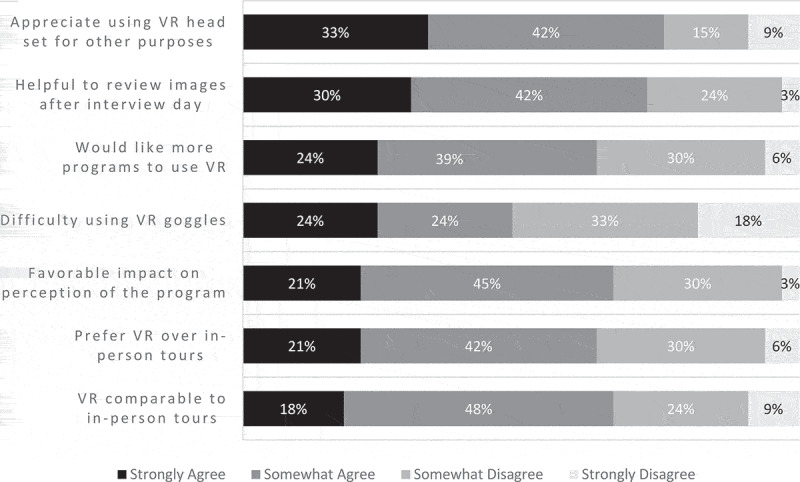
Applicant responses to survey regarding VR use during residency interview day.

The online survey was completed by 33 out of the 54 applicants who interviewed. Two-thirds of respondents (66%, n = 22) either agreed or strongly agreed that VR was a comparable substitute for in-person facility tours and 63% (n = 21) agreed or strongly agreed that they preferred VR tours over in-person facility tours. Two-thirds of respondents (66%, n = 22) also agreed or strongly agreed that the use of VR had a favorable impact on their perception of the program while 63% (n = 21) agreed or strongly agreed that they would like to see more programs using VR in the interview day. However, nearly half of the applicants (48%, n = 16) agreed or strongly agreed that they had difficulty using the VR goggles. Most applicants (72%, n = 24) agreed or strongly agreed that it was helpful to review the facility images after the interview day and 75% (n = 25) agreed or strongly agreed that they appreciated being able to use the provided VR head set for recreational purposes after the interview day (Figure 1).

The in-person tour of the primary teaching hospital rated an average 4.34 out of 5 and VR tour of the children’s hospital was rated an average 3.93 out of 5. In order of importance from 1 to 11, 1 being the most important, applicants rated several items of the interview process. The in-person facility and VR tours were ranked 8th and 9th out of 11 respectively (Table 2). Applicants provided a variety of positive and negative comments about the experience (Table 3).

**Table 3.** Applicant comments regarding their experience utilizing VR during residency interview day.

Applicant Themes and Associated Comments	
Interest in novel technology/innovation	Exciting and new technology made the day fun and interesting. Very innovative – nice way to stand out! I had never been on a Virtual Reality Tour – and found it a fun experience! I liked the novelty of it, and I appreciate the program trying something different. The VR tour was very interesting to experience, and I got a lot from that
Appreciation in decreased energy/time expenditure during interview day	Virtual tour was great! Too many walking tours this season. Very positive experience! Showed respect for our feet, but we were still able to see what it looks like. I really liked the virtual tour as it saved much time and it was cold outside!
Concern for lack of in-person view of the facilities reviewed in virtual tour	I thought it was an interesting idea; however, for this really critical decision I would have liked seeing the hospital in person. I would swap the virtual tour for a real one. While it isn’t very important to see the facilities, if you are going to promote the hospital it would be better to show it via real life tour.
Concern for difficulty with the implementation and/or utilization of novel technology	Strongly dislike. VR has not yet been made properly for people wearing contact lenses and often makes them nauseous or gives them headaches/migraines Virtual reality tour was a bit complicated; I think a PowerPoint of some images of [the children’s hospital] would be an easier alternative. I think it is a great tool, unfortunately I experienced severe motion sickness. The pictures of the facility were a sufficient substitute. I did find the VR tour useful, however it was a little challenging for some applicants to work with. Unclear if that was a malfunction/issue with the app or not. A video tour while more old school may be an alternative to consider

During the previous interview season, applicants spent 1.5 hours at the off-site children’s hospital, 1 hour in transport time, and completed the interview day by approximately 3:30 PM. During the 2018–2019 season, applicants spent 30 minutes touring the children’s hospital via VR, and of the time saved from eliminating the transport period and a second walking tour, 1 hour was used towards additional face-to-face time with residents and program leadership at an extended lunch provided at the primary hospital. The interview day was completed by 2 PM with the 2018–2019 schedule.

During the previous season, the Rutgers Med-Peds department spent 2,520 USD USD on transportation between sites. The personalized VR cardboard headsets saved 2,345 USD USD, which was then utilized to fund applicant pre-interview dinners and to support other program activities throughout the year.

## Discussion

Substituting a VR tour of the off-site children’s hospital in place of an in-person tour significantly impacted the 2018–2019 interview season by saving time and money while improving applicants’ perception of the program. The time saved by implementing the VR tour permitted more face to face time with the residents, faculty and program director, which were rated as the top 3 most important features of the interview day and allowed for earlier completion of the day by 90 minutes. While this study provided a VR experience in-person, it is also possible to provide applicants a similar experience remotely in order to save applicant time and money.

Most applicants interview at several institutions as part of the matching process, which may result in ‘interview fatigue’ as exemplified by one of the comments: ‘Virtual tour was great! Too many walking tours this season.’ This could explain why a majority of the applicants preferred VR tours over in-person tours. However, it is important to highlight that despite both tours being ranked low (positions 8 and 9 respectively) on the applicants’ list of priorities, it is difficult to assess the overall importance of having facility tours from our data. Data from an Emergency Medicine Program that surveyed over 200 applicants suggests that facility tours are the least important part of the interview day for applicants, while the overall ‘feel’ or ‘personality’ of a program is the most important. [6] On the other hand, 83% of 70 radiology applicants from a different program, viewed the tour of the primary radiology facility as necessary or desirable. [7] It is interesting to note that only 66% of the same radiology applicants felt it necessary or desirable to tour off-campus facilities. Using VR as an alternative to an in-person facility tour is beneficial for the program overall and allows for time to be spent in more ‘valuable’ parts of the interview process.

Saving 2,345 USD in recruitment funds allowed for the program’s budget to be spent on other necessary resources like applicant pre-interview dinners, in which current residents spend time with the applicants the night prior to the interview. Even more cost savings could be distributed to the applicants by allowing this technology to be used from home and avoiding travel costs all together. Especially in light of the current pandemic and recommendation from the AAMC to do all interviews ‘virtually,’ one could simply mail the google cardboard headsets to each applicant with directions on its use for a nominal postage fee. Photos and video files can be easily downloaded from any website or share drive for free and interactive tour guides can be pre-recorded or provided to the applicants through teleconferencing. It still remains to be seen whether programs throughout the country opt for a fully virtual interview process or for a mixed one, in which parts of the interview are conducted remotely but allowing applicants to also physically visit the institution. VR in either situation would allow for significant cost savings, while keeping social distancing measures in place.

The VR tour also had a favorable impact on more than half of the applicants’ perception of the program and almost as many would like to see VR used by other programs. One applicant commented, ‘Exciting and new technology made the day fun and interesting.’ The current pandemic will likely make it even harder for programs to make a significant impression through virtual media, especially if teleconferencing becomes a standardized approach. In a highly competitive environment where residency training programs try to increase their appeal to prospective residents, innovative technology, such as VR, can make a difference on the applicants’ rank lists. Incorporating VR into a program’s interview process can be a tricky process. As nearly half of the applicants had difficulty using the goggles, it is necessary to have dedicated staff or expertise available to the applicants to help troubleshoot the process. The ease of the VR tour was largely reliant on the applicant’s ability to download the app and photos to their smart phones prior to the interview day, and there seemed to be a large variability of app functionality and phone quality. Some of the comments from the applicants showed how technically challenging the process can be, even in person, and suggest that a poor implementation risks a detrimental impact to the applicant’s impression of the program. Purchasing headsets that include a screen and have the images pre-loaded could overcome these technical difficulties, however, would increase the cost of the technology and would unlikely be offered remotely. Some individuals even experienced motion sickness while using the headset. Having an alternative for the VR tour such as a slide show of images from the off-site hospital should be an alternative for applicants who are unable to tolerate VR. None-the-less, this program’s experience can assist those wishing to adapt the technology to their own needs. The implementation of VR for hospital tours has the potential of becoming common place during the current pandemic and, if done well, can make a program stand-out.

It will need to be determined by each program how essential the hospital tour is to the overall application process considering that our applicants ranked facility tours low on the list of interview components. Due to the pandemic, losing face time with residents and faculty could make the facility tour more important to the applicants. This pilot study suggests that VR facility tours have the potential of replacing in-person tours at alternate hospital sites, but a direct comparison between a VR tour done remotely versus in-person of the same site would remain to be validated. Even though 66% of the applicants either somewhat or strongly agree that VR tours are comparable to in-person ones, the in-person tour was rated higher than the VR tour (4.34 vs 3.93), however, these results are difficult to interpret since they evaluate different facilities (main hospital vs alternate hospital). This lack of a ‘randomized-control’ trial limits this study’s ability to directly compare the quality of the in-person tour of the primary teaching site versus the VR tour of the children’s hospital. Performing such a study would require randomizing applicants to either an in-person tour group or a VR tour group of the same site, which would be hard to accomplish during an interview season. Studies would also need to compare the effectiveness of doing VR tours remotely versus receiving VR instruction in-person.

## Conclusion

Use of VR facility tours as an alternative to in-person tours of off-site teaching hospitals during a residency interview day is a viable and innovative option that can save time and money, and favorably impact the applicant’s impression of the program. More research is necessary to assess whether VR tours can replace in-person tours at the main hospital teaching site. However, as parts of the interview process become remote to comply with social distancing requirements and recommendations, having the experience and infrastructure to implement VR tours might become necessary for programs moving forward.
